# Oversimplification of a complex public health issue that serves exploitative industry interests

**DOI:** 10.1111/add.70090

**Published:** 2025-05-08

**Authors:** Raglan Maddox, Andrew Waa, Tom Calma, Lisa J Whop

**Affiliations:** ^1^ Bagumani (Modewa) Clan, Yardhura Walani, National Centre for Aboriginal and Torres Strait Islander Wellbeing Research, National Centre for Epidemiology and Population Health, Australian National University (ANU) Canberra Australia; ^2^ Ngāti Hine/Ngāpuhi. Eru Pomare Māori Health Research Unit University of Otago Wellington New Zealand; ^3^ University of Sydney Sydney Australia; ^4^ Wagadagam. Yardhura Walani, National Centre for Aboriginal and Torres Strait Islander Wellbeing Research. National Centre for Epidemiology and Population Health Australian National University (ANU) Canberra Australia

**Keywords:** base rate fallacy, e‐cigarettes, equity, Indigenous, tobacco, tobacco control, tobacco industry, vaping, youth nicotine use

Mendelsohn *et al*. [1] suggest Aotearoa/New Zealand's rapid smoking decline is because of less restrictive vaping policies, yet this claim is based on flawed assumptions and weaknesses in methods [2, 3]. The article selectively reports data to support pro‐vaping positions [4, 5, 6, 7], warranting caution from readers.

Extrapolating Aotearoa/New Zealand's smoking trend pre‐2019 to 2023 suggests only a 3% additional decline beyond the existing trajectory. Mendelsohn *et al*. [1] inflate vaping's role by downplaying pre‐existing smoking declines. They highlight Māori smoking reductions (35.5%–17.1%), but omit evidence of prior declines before widespread vape adoption [1].

Further, the study uses an unverified assumption to estimate Australia's 2016 smoking prevalence, inflating Aotearoa/New Zealand's comparative decline. The authors also fail to acknowledge vaping industry lobbying and the repeal of world‐leading tobacco policies [8, 9], instead framing vaping as indispensable to tobacco control.

Mendelsohn *et al*. [1] also commit a base rate fallacy [2], asserting: ‘The potential harm from additional vaping needs to be balanced against the likelihood that vaping is diverting some young people who would otherwise have smoked away from cigarettes.’ This ignores the steady decline in youth smoking before vapes entered the market and the simultaneous surge in nicotine uptake among youth who never smoked (Figures 1, 2, 3, 4) [10]. Rather than accelerating declines, vaping coincides with a flattening of smoking reductions.

**FIGURE 1:**
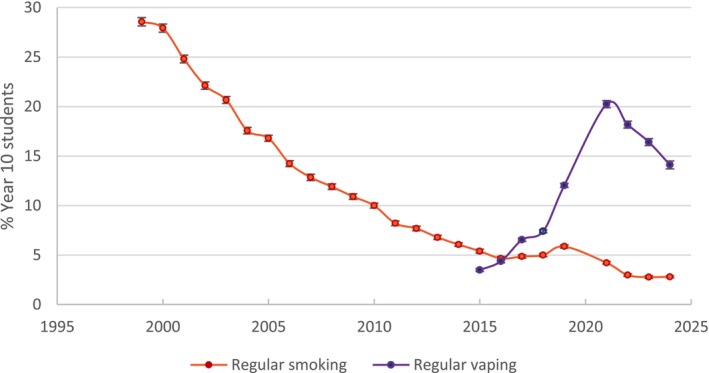
Regular smoking prevalence (1999–2024) and regular vaping (2015–2024) for year 10 students (14–15 years).

**FIGURE 2:**
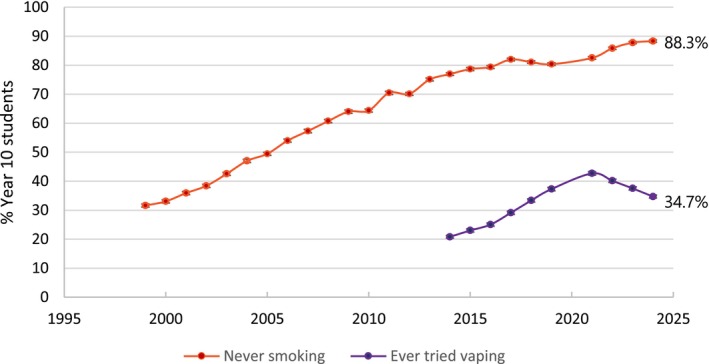
Never smoking prevalence (1999–2024) and ever tried vaping (2015–2024) for year 10 students (14–15 years).

**FIGURE 3:**
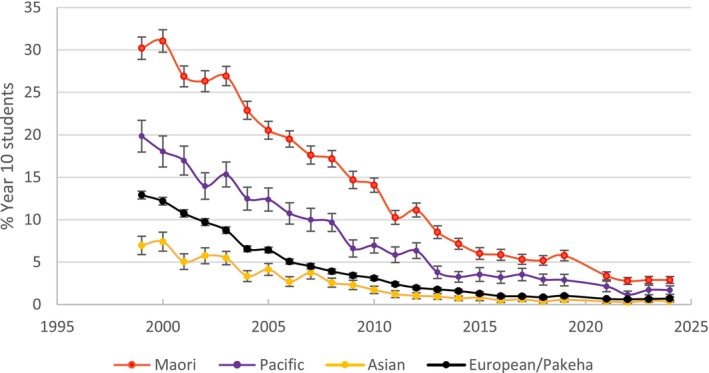
Daily smoking prevalence by ethnicity (1999–2024) for year 10 students (14–15 years).

**FIGURE 4:**
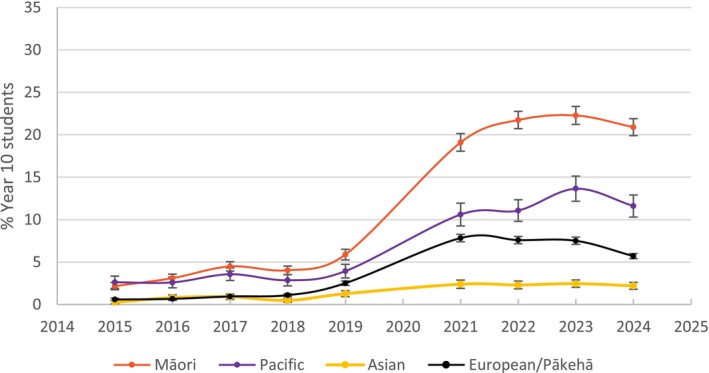
Daily vaping prevalence by ethnicity (2015–2024) for year 10 students (14–15 years).

Mendelsohn *et al*. [1] imply Aotearoa/New Zealand's smoking decline is because of permissive vaping policies, despite briefly acknowledging this cannot be proven [1]. However, they fail to adequately consider alternative factors—tobacco control policies, enforcement, economic conditions and public health initiatives [1, 8, 11, 12]. This misrepresents correlation as causation [2, 3].

Mendelsohn *et al*. [1] use selective framing reinforces an industry viewpoint, neglecting alternative explanations [3]. The failure to adjust for pre‐existing smoking trends is a major methodological flaw [1, 3].

Mendelsohn *et al*. [1] selectively cite high smoking rates among Indigenous peoples to justify vaping policies without acknowledging the disproportionate harm of rising vaping rates [12, 13, 14, 15]. Among Māori youth, daily vaping rates now exceed historical smoking rates—contradicting claims that vaping is a cessation tool [9, 10]. This omission perpetuates a neo‐colonial approach that prioritises corporate interests over Indigenous sovereignty [1, 16, 17].

In summary, Mendelsohn *et al*. [1] misrepresent causation and downplay vaping‐related harms [9, 13]. Public health efforts should prioritise Indigenous‐led commercial tobacco resistance strategies, strengthen regulation and eliminate industry interference [18].

## AUTHOR CONTRIBUTIONS

**Raglan Maddox:** Conceptualization (equal); data curation (equal); investigation (equal); methodology (equal); supervision (equal); validation (equal); writing—review and editing (equal). **Andrew Waa:** Conceptualization (equal); data curation (equal); formal analysis (equal); investigation (equal); methodology (equal); project administration (equal); supervision (equal); validation (equal); writing—original draft (equal); writing—review and editing (equal). **Tom Calma:** Conceptualization (equal); formal analysis (equal); investigation (equal); methodology (equal); supervision (equal); writing—review and editing (equal). **Lisa J Whop:** Conceptualization (equal); formal analysis (equal); investigation (equal); methodology (equal); project administration (equal); supervision (equal); validation (equal); writing—review and editing (equal).

## DECLARATION OF INTERESTS

None.

## Data Availability

The data that support the findings of this study are publicly available.
